# Comparative analyses of plastid genomes from fourteen Cornales species: inferences for phylogenetic relationships and genome evolution

**DOI:** 10.1186/s12864-017-4319-9

**Published:** 2017-12-08

**Authors:** Chao-Nan Fu, Hong-Tao Li, Richard Milne, Ting Zhang, Peng-Fei Ma, Jing Yang, De-Zhu Li, Lian-Ming Gao

**Affiliations:** 1 Key Laboratory for Plant Diversity and Biogeography of East Asia, Kunming Institute of Botany, Chinese Academy of Sciences, Kunming, 650201 China; 2 University of Chinese Academy of Sciences, Beijing, 100049 China; 3 Germplasm Bank of Wild Species in Southwest China, Kunming Institute of Botany, Chinese Academy of Sciences, Kunming, 650201 China; 4 Institute of Molecular Plant Sciences, University of Edinburgh, King’s Buildings, Edinburgh, Scotland EH9 3JH UK

**Keywords:** Plastid genome, Phylogenomics, Cornales, Alignment, Partitioning schemes, Gene loss

## Abstract

**Background:**

The Cornales is the basal lineage of the asterids, the largest angiosperm clade. Phylogenetic relationships within the order were previously not fully resolved. Fifteen plastid genomes representing 14 species, ten genera and seven families of Cornales were newly sequenced for comparative analyses of genome features, evolution, and phylogenomics based on different partitioning schemes and filtering strategies.

**Results:**

All plastomes of the 14 Cornales species had the typical quadripartite structure with a genome size ranging from 156,567 bp to 158,715 bp, which included two inverted repeats (25,859–26,451 bp) separated by a large single-copy region (86,089–87,835 bp) and a small single-copy region (18,250–18,856 bp) region. These plastomes encoded the same set of 114 unique genes including 31 transfer RNA, 4 ribosomal RNA and 79 coding genes, with an identical gene order across all examined Cornales species. Two genes (*rpl22* and *ycf15*) contained premature stop codons in seven and five species respectively. The phylogenetic relationships among all sampled species were fully resolved with maximum support. Different filtering strategies (none, light and strict) of sequence alignment did not have an effect on these relationships. The topology recovered from coding and noncoding data sets was the same as for the whole plastome, regardless of filtering strategy. Moreover, mutational hotspots and highly informative regions were identified.

**Conclusions:**

Phylogenetic relationships among families and intergeneric relationships within family of Cornales were well resolved. Different filtering strategies and partitioning schemes do not influence the relationships. Plastid genomes have great potential to resolve deep phylogenetic relationships of plants.

**Electronic supplementary material:**

The online version of this article (10.1186/s12864-017-4319-9) contains supplementary material, which is available to authorized users.

## Background

The Cornales is a relatively small but diverse group, representing the basal lineage of the largest angiosperm clade, the Asterids [1–4]. It comprises 42 genera and approximately 605 species in ten families, including two large families (Hydrangeaceae and Loasaceae) and eight small families. The latter contain few genera, mostly with isolated geographic ranges, i.e. Cornaceae (*Cornus*), Nyssaceae (*Camptotheca*, *Nyssa*), Curtisiaceae (*Curtisia*), Grubbiaceae (*Grubbia*), Hydrostachyaceae (*Hydrostachys*), Alangiaceae (*Alangium*), Davidiaceae (*Davidia*), and Mastixiaceae (*Diplopanax*, *Mastixia*) [3, 5–8]. Cornales contains many ecologically and economically important species, including ornamentals in Cornaceae, Davidiaceae and Hydrangeaceae; moreover Camptotheca (Nyssaceae), is the source of camptothecin. Species in the order possess different habits (evergreen, deciduous), diverse growth forms (e.g. trees, shrubs, lianas, rhizomatous and herbs) and occur in tropical, temperate and boreal ecosystems.

The circumscription and phylogenetic relationships of the order have been investigated by a number of phylogenetic analyses, mostly based on plastid DNA, beginning from the early twentieth century such as Olmstead et al. [9] and Chase et al. [10]. Increasing the amount of molecular markers has progressively improved phylogenetic resolution and branch support in Cornales [3, 5, 7, 8]. For example, based on six cpDNA regions and broader taxon sampling, Xiang et al. [3] obtained well supported but not fully resolved intra-family relationships for some families (e.g. Hydrangeaceae, Cornaceae) in this order.

Integrating genomic data into plant phylogenetic investigations is developing rapidly due to the availability of new methods of sampling genomes (e.g. genome skimming, transcriptomes, hybrid capture) facilitated by next-generation sequencing (NGS) technologies [11–14]. Complete plastid genomes have rapidly accumulated in the NCBI databases over the last few years. However, phylogenomic studies remain to be conducted for Cornales, and only a few plastid genomes within this order have been released, sporadically, on NCBI databases. The plastome is usually uniparentally inherited in seed plants [15]. It can provide an abundance of variable sites across its entire length for phylogenetic analyses [16]. Thus, plastid genomes show the potential for resolving recalcitrant phylogenetic relationships, at both high taxonomic levels such as green plants [17–19], and low taxonomic levels [20–22]. The most widely used approach for plastome phylogenomics is to analyze the concatenated coding genes as a whole [14, 20, 23, 24], but the noncoding sequences are also useful for inferring phylogenies at lower taxonomic levels when the plastid genomes are conserved [25–27]. Because different regions of plastid genomes vary in their evolutionary rates, partitioning the genome by genes or regions might be preferable for phylogenomic analysis [20]. Moreover, fast-evolving sites of plastid genomes that cannot be aligned with confidence could possibly result in misleading phylogenetic inference, and therefore removing the most variable sites or problematic regions might improve accuracy in phylogenetic inference [28–30].

Plastid genomes of angiosperms generally contain 110 to 130 distinct genes, and range in size from 120 to 160 kb. They usually show a typical quadripartite circular structure of two copies of large inverted repeat (IR) separated by the small single-copy (SSC) and the large single-copy (LSC) regions [31]. Although the plastid genome is reported as highly conserved in most angiosperms [32], it is subject to structural alterations such as extension or contraction of the IR region [33], the presence of large inversions or deletions [34, 35], pseudogenization and gene loss [36, 37]. Besides their phylogenetic utility, whole plastid genomes could also be used to investigate other aspects of molecular biology such as genome evolution on the structural and molecular level, and to develop fast evolved molecular markers for investigations of phylogeny and phytogeography [17, 27, 38–41].

In the present study, a total of 15 complete plastid genomes of 14 species representing 10 genera and seven families of Cornales were obtained. The main objectives of this study were to 1) characterize and compare the structure and gene organizations of plastid genomes in Cornales; 2) explore the potential effects of different partitioning schemes and alignment strategies on phylogenetic inferences; and 3) assess the application of the complete plastid genome sequences in Cornales, and provide genetic resources for future research.

## Methods

### Taxon sampling

The circumscription of families of Cornales followed Xiang et al. [3], and taxonomy within families and genera followed Flora of China [42] or the Plant List (http://www.theplantlist.org/) (accessed 1st January, 2013). A total of 15 individuals representing 14 species of 10 genera from 7 families in Cornales mainly occurring in China were sampled. Samples of three families (Grubbiaceae, Hydrostachyaceae and Loasaceae) could not be obtained for this study. The sampled species hence represented four out of the five major lineages suggested by Xiang et al. [3]. Two individuals of *Cornus capitata* were sequenced here to investigate the intraspecific variability within plastid genome. As outgroups, the plastid genome of *Fouquieria diguetii* of Ericales was newly sequenced, and the plastomes of three species within Caryophyllales (*Basella alba*, *Talinella dauphinensis*, *Gisekia pharnaceoides*) were obtained from another parallel work (unpublished data). These two orders are phylogenetically closest to Cornales [1–4]. Fresh leaves were collected in the field or from botanic gardens with the permission of the land owners or the botanic gardens (Table 1) and transferred to the laboratory under cool conditions (~4 °C) for total genomic DNA extraction. Voucher specimens were collected for each species, and deposited at the Herbarium of Kunming Institute of Botany (KUN), Chinese Academy of Sciences or the herbarium of the Royal Botanic Garden Edinburgh (E). Detailed information of the pecies sampled in this study is provided in Table 1.

**Table 1:** Taxa sampled in this study

Taxa	Family	Order	Locality	Voucher	Voucher specimen	GenBank accession number
*Nyssa wenshanensis*	Nyssaceae	Conales	China, Yunnan, Kunming Botanical Garden	Cai J. & Zhang T.	14CS9047	MG524995
*Nyssa sinensis*	Nyssaceae	Conales	China, Yunnan, Wenshan	Liu C., et al.	14CS8436	MG525000
*Camptotheca acuminata*	Nyssaceae	Conales	China, Yunnan, Yuxi	Cai J., et al.	13CS7273	MG525005
*Davidia involucrata*	Davidiaceae	Conales	China, Yunnan, Kunming Botanical Garden	Cai J. & Zhang T.	14CS9049	MG525002
*Mastixia caudatilimba*	Mastixiaceae	Conales	China, Yunnan, Xishuangbannan,	Guo Y.J., et al.	14CS9459	MG525001
*Diplopanax stachyanthus*	Mastixiaceae	Conales	China, Yunnan, Wenshan,	Zhang T. & Liu C.	14CS8795	MG524991
*Hydrangea heteromalla*	Hydrangeaceae	Conales	China, Yunnan, Kunming	Guo Y.J. & Liu C.	10CS1923	MG524994
*Hydrangea aspera*	Hydrangeaceae	Conales	China, Yunnan, Wenshan	Liu C., et al.	14CS8432	MG524992
*Deutzia crassifolia*	Hydrangeaceae	Conales	China, Yunnan, Chuxiong	Guo Y.J., et al.	14CS8216	MG524993
*Alangium alpinum*	Alangiaceae	Conales	China, Yunnan, Kunming Botanical Garden	Yang J.D.	14CS9086	MG525003
*Alangium chinense*	Alangiaceae	Conales	China, Yunnan, Wenshan	Cai J., et al.	14CS9130	MG524996
*Cornus capitate #1*	Cornaceae	Conales	China, Yunnan, Kunming	Ya J.D., et al.	14CS9213	MG524990
*Cornus capitate #2*	Cornaceae	Conales	China, Yunnan, Kunming Botanical Garden	Liu C. & Ya J.D.	14CS8464	MG524998
*Cornus controversa*	Cornaceae	Conales	China, Yunnan, Kunming Botanical Garden	Liu C. & Ya J.D.	14CS8466	MG525004
*Curtisia dentata*	Curtisiaceae	Conales	UK, Royal Botanic Garden Edinburgh	Möller M.	RBGE 19240177	MG524999
*Fouquieria diguetii*	Fouquieriaceae	Ericales	UK, Royal Botanic Garden Edinburgh	Möller M.	RBGE 19800074	MG524997
*Basella alba*	Basellaceae	Caryophyllales	China, Yunnan, Kunming	Yang J.D. et al.	14CS9526	Unpublished
*Talinella dauphinensis*	Talinaceae	Caryophyllales	UK, Royal Botanic Garden, Kew	Yi T.S.	Yi14363	Unpublished
*Gisekia pharnaceoides*	Gisekiaceae	Caryophyllales	China, Hainan, Lingshui,	Zhang T., et al.	14CS8741	Unpublished

### DNA sequencing and genome assembly

Total genomic DNA was isolated from about 100 mg fresh leaf material with a modified CTAB method [43] in which 4% CTAB was used instead of 2% CTAB and with approximately 0.1% DL-dithiothreitol (DTT) added. Subsequently, plastid DNA was selectively amplified through long-range PCR using nine or fifteen primer pairs [44, 45]. All PCR products were pooled and diluted to 0.2 ng/μL for library preparation. A short-insertion (500 bp) sequencing library was prepared following the Nextera XT Sample Preparation procedure (Illumina). The paired-end reads of 250 bp or 300 bp were generated using Illumina Miseq at the Laboratory of Molecular Biology of Germplasm Bank of Wild Species, Kunming Institute of Botany, Chinese Academy of Sciences. Four species could not be amplified through long range PCR: *Camptotheca acuminata*, *Cornus controversa*, *Mastixia caudatilimba* and *Hydrangea heteromalla*. These were sequenced instead from total DNA using Illumina Hiseq4000 after short-insert (500 bp) libraries constructed following the manufacturer’s protocol (Illumina HiSeq 4000) and 143 bp paired-end reads for *Hydrangea heteromalla* and 90 bp paired-end reads for the other three species, which were generated at BGI Shenzhen, China.

The raw sequence reads were assembled using following steps. First, all reads were de novo assembled into contigs with CLC Genomics Workbench 8.0.2 (CLC Bio) under a word size of 60 bp, minimum contig length of 500 bp and map reads back to contigs with default settings. Second, a closely related genome of *Camellia sinensis* (NC_020019.1) was used as a reference, and contigs of each individual sample were aligned to it using local BLAST, from which the contigs of plastid genome can be selected. For *Cornus controversa* and *Hydrangea heteromalla,* this process produced two and three long plastid contigs respectively, which were easily assembled into a complete genome by overlaps using Geneious v 8.1 [46]. Among the remaining 14 samples, parts of the genome were covered only by short contigs, which were hard to assemble directly. These were analyzed using the two successfully assembled species as reference sequences, and then manually concatenated by their overlaps in Geneious v 8.1.

Verification of the assembly was performed in three ways: 1) by mapping the reads to the assembled plastid genome sequences, 2) by comparing the 14 manually assembled genomes with two easily assembled ones, and 3) by obtaining the four boundary regions using newly designed primers under Sanger sequencing, which were showed in Additional file 1: Table S1.

### Genome annotation and comparison

The complete genome sequences were annotated using the online program DOGMA [47] to predict protein-coding genes, transfer RNA (tRNA) genes, and ribosome RNA (rRNA) genes. Start and stop codons of protein-coding genes were determined using plastid/bacterial genetic codes, with the most closely matching reference genome as a guide. Graphical maps with annotation of genomes were drawn using OrganellarGenomeDRAWtool (OGDraw) [48].

The 15 whole plastid genomes were aligned with Mauve v 2.3.1 [49] plugin in Geneious v 8.1, including only one copy of the IR, assuming collinear genomes for the full alignment. To compare the overall similarities among different plastid genomes, pairwise alignments of the 15 genomes of Cornales were performed in the mVISTA program [50], under LAGAN mode using the annotations of *Cornus controversa* as reference. Plastomes of Cornales were also aligned using MAFFT [51] and manually edited in Geneious v 8.1. To observe the plastid genome divergence and determine parsimony informative sites, sliding window analysis was conducted after alignment. In order to identify some mutational hotspots, the proportion of mutational events was calculated following a modified version of the formula used by Gielly and Taberlet [52]: the proportion of mutation events = [(NS + ID)/L] * 100%, where NS is the number of nucleotide substitutions, ID is the number of indels and L is the aligned sequence length of each region. Hotspots were here defined as those regions with a value >20%. The step size was set to 200 bp, with a 600 bp window length as described by Xu et al. [27].

To test whether the abnormal gene of *rpl22* is disabled or not, the ratio of nonsynonymous and synonymous (ω, d_N_/d_S_) of *rpl22* for different branches was calculated in PAML v4.7 [53] using the codeml module.

### Alignment and subdivision of plastid genomes

The whole plastid genomes of the 15 individuals of Cornales and the four outgroup species were aligned using the program MAFFT v 7.22 with default settings. Three primary data sets were generated for phylogenetic inference. The first data set comprised coding regions, i.e. exons of protein-coding genes, tRNAs and rRNAs; the second comprised all noncoding regions, i.e. intergenic regions and introns; the third comprised the entire plastid genome. Each gene and intergenic or intron was realigned using MAFFT v 7.22 with G-INS-i algorithm plugin in Geneious v 8.1. One of the IR regions was removed for all data sets to reduce overrepresentation of duplicated sequences.

Some regions in the whole plastome data set are highly variable and poorly aligned. So, in order to assess the effect of alignment quality on phylogeny, we compared the results from three different analysis strategies. First, the unfiltered alignment included all sequence positions of the plastomes in the alignment. Second, the lightly filtered alignment was created using the program Gblocks [54] to remove those regions that were identified as highly variable or ambiguously aligned, using the program’s default parameters; only positions where 50% or more of the sequences had a gap were retained. Third, the strictly filtered alignment was generated using the same approach as the lightly filtered alignment, but excluding all those positions that had at least one gap.

### Phylogenetic analyses

For the unfiltered, lightly filtered and strictly filtered alignments of coding, noncoding and complete plastome data sets, jModeltest v2.1.6 [55] was used, as implemented on the Cyberinfrastructure for Phylogenetic Research (CIPRES) cluster (http://www.phylo.org/), to estimate the optimal model of molecular evolution with the Akaike Information Criterion (AIC). Maximum likelihood (ML) analyses were conducted using RAxML v8.1.11 [56] as implemented on the CIPRES cluster. These RAxML searches relied on the general time reversible model of nucleotide substitution, with the gamma model of rate heterogeneity (GTR + G) as suggested (see RAxML manual). The ML trees were inferred using the rapid bootstrap with 1000 replicates, and the best-scoring ML tree was sought. Bayesian inference (BI) analyses were conducted with MrBayes v3.2.3 [57] as implemented on the CIPRES cluster with the models estimated for the different data sets (Additional file 1: Table S2). Two runs were conducted in parallel with four Markov chains (one cold and three heated), with each running for 2,000,000 generations from a random tree and sampled every 100 generations. The convergence was checked using the average standard deviation of split frequencies (ASDFs) (<0.01). The first 25% of the trees were discarded as burn-in, and the remaining trees were used to construct majority-rule consensus trees.

To investigate the issues of data partitioning for the plastid phylogenomic analysis, an algorithmic method for estimating an optimal partitioning scheme was conducted for the complete unfiltered data set. It was partitioned into the maximum possible number of data blocks based on genomic composition. We divided the whole plastid genome into 174 subsets: each gene, intergenic region or intron was regarded as a distinct subset, while subsets of less than 200 bp, or regions that only contained invariable nucleotide sites, were combined into large data subsets according to their function (see details in Additional file 1: Table S3). Subsequently, the program PartitionFinder v1.1.1 [58] was used to identify the best partitioning schemes of these 174 subsets according to the Bayesian information criterion (BIC) using a heuristic search (search = rcluster).

For partitioned ML phylogenetic analysis, a partitioned model was used to specify the regions of alignment, for which an individual model of nucleotide substitution was estimated. Individual per-partition branch lengths were estimated using RAxML v8.1.11 software. For partitioned BI phylogeny estimation, each partition was given its own optimal model (GTR + G or GTR + G + I) (Additional file 1: Table S3). All parameters were set to be unlinked across partitions except those for branch lengths and topology; branch length rate multipliers were unlinked in MrBayes v3.2.3.

## Results

### Characteristics of the plastid genomes

**Fig. 1:**
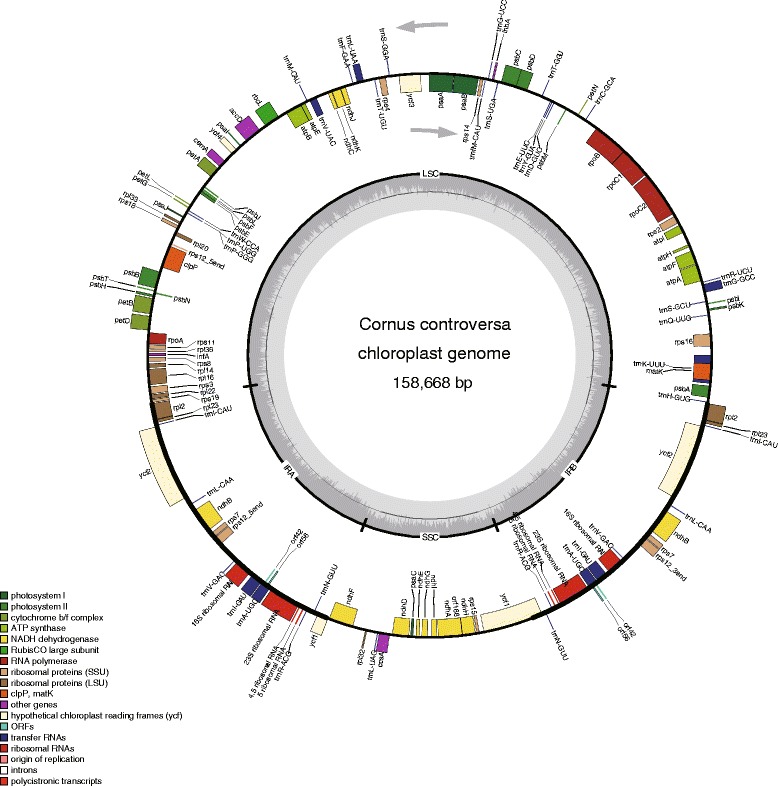
Gene map of *Cornus controversa* as a representative of Cornales. Genes on the inside of the outer circle are transcribed clockwise and those outsides are transcribed counterclockwise. Functional categories of genes are color-coded. The dashed area in the inner circle indicates the GC content of the plastid genome

Fifteen complete plastid genomes of Cornales, plus *Fouquieria diguetii* of Ericales were newly generated in this study; these genome sequences have been submitted to GenBank (Table 1). The mean coverage depth of these plastomes ranged from 383× (*Alangium chinense*) to 2757× (*Camptotheca acuminata*). Henceforth, all text describing plastid genomes refers only to Cornales unless stated otherwise. The size of the 15 Cornales plastid genomes ranged from 156,567 bp in *Nyssa sinensis* to 158,715 bp in *Diplopanax stachyanthus*, and both individuals of *Cornus capitata* examined had the same plastid genome size (157,200 bp) (Table 2). All of the 16 sequenced plastid genomes displayed a typical quadripartite structure (Fig. 1), comprising a pair of IRs (25,859–26,451 bp) separated by the LSC (86,089–87,835 bp) and the SSC (18,250–18,856 bp) regions (Table 2). The LSC regions exhibited the greatest standard deviation in sequence length (s.d. = 586 bp), followed by SSC regions (s.d. = 188 bp) and the IR regions (s.d. = 147 bp). The full genomes encoded 114 unique genes, which included 31 tRNA genes, four rRNA genes and 79 protein-coding genes with the same gene order. There were 16 genes duplicated in the IR regions, resulting in a total of 130 genes (Additional file 1: Table S4). Seventeen of those genes contained one intron, and two genes (*ycf3* and *clpP*) contained two introns. The length and GC content of coding, noncoding and complete plastid genome data sets are shown in Table 2. Noncoding regions (s.d. = 789 bp) showed more variation in sequence length than coding regions (s.d. = 115 bp). Among Cornales species, the percentage of the coding regions varied from 57.2% to 58.2%. The overall GC content is similar across individuals in coding and noncoding regions of Cornales, but a little higher than that of *Fouquieria diguetii* of Ericales (Table 2).

**Table 2:** The plastid genome features of the sequenced species

*Taxon*	Full		LSC length (bp)	SSC length (bp)	IR length (bp)	Gene Number	Protein-coding	RNAs	Coding region		Noncoding region		Mean Coverage
	Length (bp)	GC (%)							Length (bp)	GC (%)	Length (bp)	GC	
*Nyssa wenshanensis*	156,598	37.9	86,109	18,261	26,114	114	79	35	91,073	40.3	65,525	34.6	974
*Nyssa sinensis*	156,567	37.9	86,089	18,250	26,114	114	79	35	91,073	40.3	65,494	34.6	802
*Camptotheca acuminata*	157,811	37.8	87,333	18,760	25,859	114	79	35	91,078	40.3	66,772	34.4	2757
*Davidia involucrata*	158,131	37.8	87,335	18,856	25,970	114	79	35	91,037	40.3	67,094	34.4	1026
*Mastixia caudatilimba*	158,221	37.8	87,418	18,797	26,003	114	79	35	90,962	40.3	67,259	34.4	1889
*Diplopanax stachyanthus*	158,715	37.8	87,679	18,632	26,202	114	79	35	90,944	40.2	67,771	34.6	1758
*Hydrangea heteromalla*	157,889	37.8	86,907	18,738	26,122	114	79	35	91,138	40.1	66,751	34.7	937
*Hydrangea aspera*	157,637	37.8	86,815	18,646	26,088	114	79	35	91,189	40.2	66,448	34.5	766
*Deutzia crassifolia*	157,035	37.6	86,583	18,714	25,869	114	79	35	91,099	40.1	65,936	34.1	549
*Alangium alpinum*	156,673	37.7	86,181	18,592	25,950	114	79	35	90,842	40.2	65,831	34.2	2121
*Alangium chinense*	156,684	37.7	86,185	18,603	25,948	114	79	35	90,824	40.2	65,860	34.2	383
*Cornus capitate #1*	157,200	38.2	86,564	18,412	26,112	114	79	35	90,928	40.5	66,272	35.0	1068
*Cornus capitate #2*	157,200	38.2	86,564	18,412	26,112	114	79	35	90,928	40.5	66,272	35.0	2523
*Cornus controversa*	158,668	37.8	87,835	18,705	26,064	114	79	35	90,823	40.4	67,845	34.3	573
*Curtisia dentata*	158,548	37.7	87,158	18,490	26,450	114	79	35	91,018	40.2	67,530	34.3	538
*Fouquieria diguetii*	157,895	37.3	87,321	18,482	26,046	114	79	35	91,244	39.9	66,651	33.7	1195

**Fig. 2:**
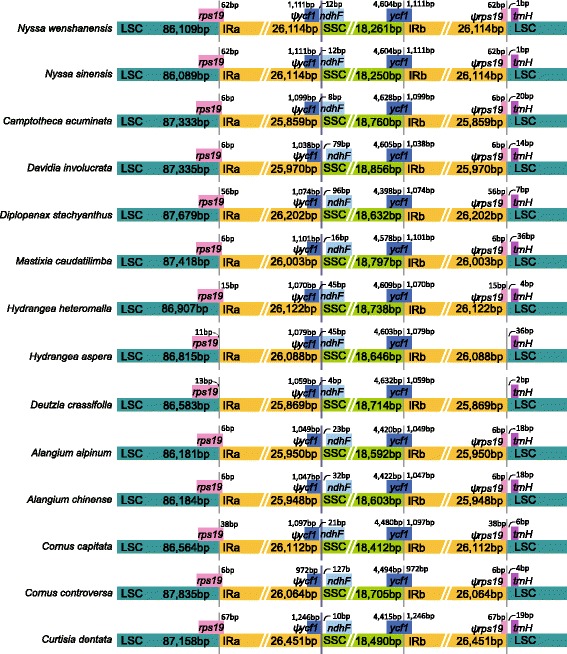
Sliding window analysis of the whole chloroplast genomes of Cornales taxa

Boundaries between the IR and SSC/LSC regions were verified by Sanger sequencing; the results were identical with the NGS sequencing. Variation in the positions of the boundaries between IR and SSC/LSC are usually considered to be the primary mechanism causing length variation among the plastid genomes of higher plants (Kim and Kim, [59]), but only slight variation was detected within Cornales (Fig. 2). The IRa/LSC junction was located within the *rps19* gene in all but two species (*Hydrangea davidii* and *Deutzia crassifolia*), resulting in the presence of a part of the *rps19* gene in the IRb. In *Hydrangea davidii* and *Deutzia crassifolia*, the junction was located in the *rps19*-*rpl2* spacer. The IRb/SSC boundary positions in all species were located in the *ycf1* gene, with part of this gene duplicated from 972 to 1246 bp. The *ndhF* gene in seven species was completely located in the SSC region, whereas in the others it extended fractionally into the IRa region (Fig. 2).

### Genome sequence divergence among Cornales

The plastid genomes within Cornales showed high sequence similarities with identities of only a few regions below 90% (Additional file 2: Fig. S1), suggesting a high conservatism of plastid genomes within Cornales. The IR regions and coding regions were more conserved than the single-copy regions and noncoding regions (Additional file 2: Fig. S1).

**Fig. 3:**
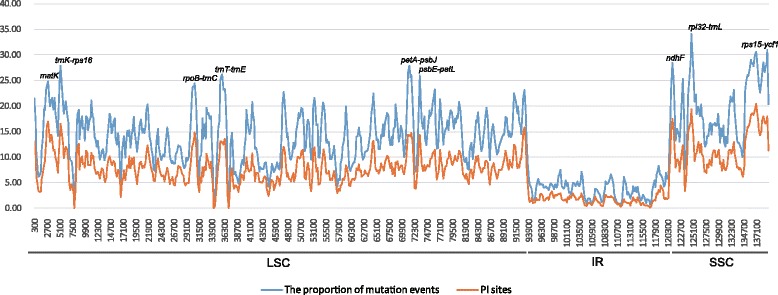
Comparison of the LSC, IR and SSC borders of 13 species of Cornales plastid genomes

Slide window analysis also showed much higher proportions of both mutation events and parsimony-informative sites in single-copy regions than in the IR region. From this, nine relatively highly variable regions (mutational hotspots) were identified from the plastid genomes, which might be undergoing more rapid nucleotide substitution. These comprised 2 gene regions and 7 intergenic regions: *matK*, *ndhF*, *trnK-rps16*, *rpoB-trnC*, *trnT-trnE*, *petA-psbJ*, *psbE-petL*, *rpl32-trnL*, and *rps15-ycf1* regions (Fig. 3). These regions are potential molecular markers for application in phylogeny and phytogeography investigations.

### Internal stop codons and putative loss of gene function

The genes *rpl22* and *ycf15* were interrupted by internal stop codons in seven and five Cornales species respectively. Both of them were further verified by Sanger sequencing using newly designed primers (Additional file 1: Table S1); the results were identical to the NGS-based plastid genome sequences.

**Fig. 4:**
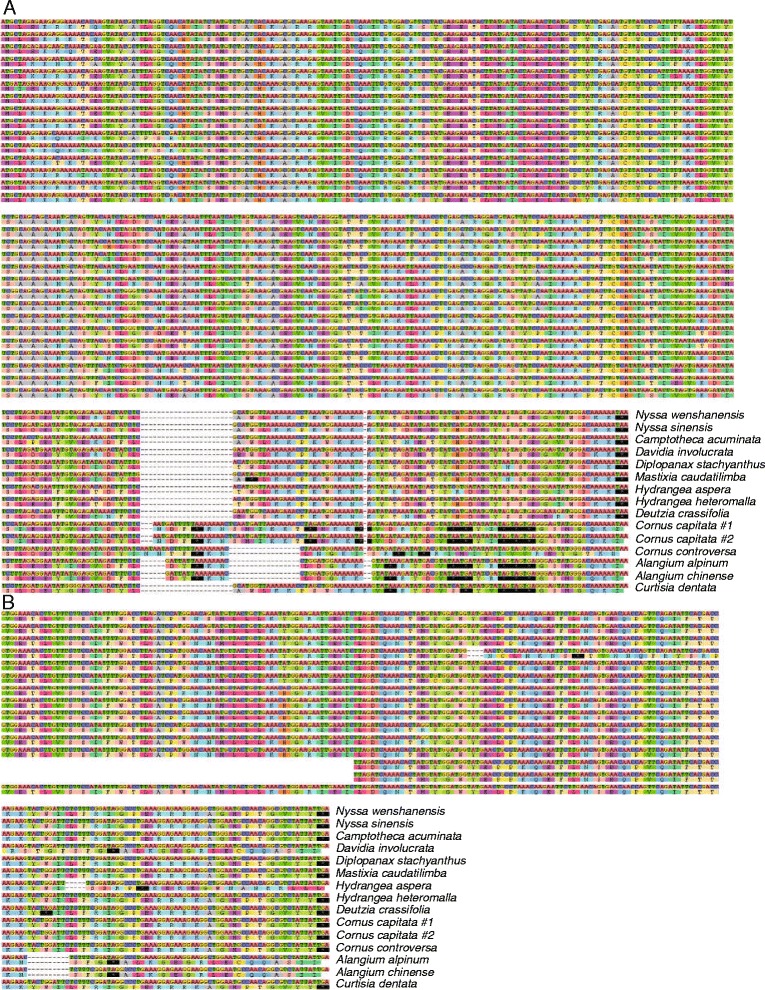
Alignment of two abnormal genes among Cornales and outgroups. **a** *rpl22* gene; (**b**) *ycf15* gene

For all species from Cornaceae, Alangiaceae and Curtisiaceae, a frameshift mutation generated premature termination codons within *rpl22*. Furthermore, *Mastixia caudatilimba* had one base change from G to A within *rpl22*, resulting in an internal termination codon (TGG to TGA) (Fig. 4a). Furthermore, this gene had a 19-bp and 5-bp insertion in *Cornus capitata* and *C. controversa* respectively, plus a 1 bp deletion in both *Alangium* species, and a 1 bp insertion in *Curtisa dentata,* all occurring upstream of the internal stop codon (Fig. 4a). *rpl22* was found to be truncated in some species, with considerable length variation (384 bp to 474 bp). Despite this, the gene still exhibited nearly 80% nucleotide identities between species, with no big difference between those species with and those without internal stop codons. Furthermore, the ratio of nonsynonymous and synonymous (ω, d_N_/d_S_) of *rpl22* for different branches showed similar values in both the Cornaceae-Alangiaceae-Curtisiaceae clade (ω = 0.34569) and Mastixiaceae clade (ω = 0.35594), and no significant difference with background (ω = 0.36549, *P* > 0.33) was found. This indicated that those genes containing stop codons have not accumulated mutations at an increased rate, and hence may not have lost their functions.

The gene *ycf15* varied from 102 bp to 249 bp among the 15 sequenced individuals of Cornales. For two species of *Alangium, ycf15* contained a large deletion (84 bp) at the 5′ end and a 10-bp deletion near the 3′ end, potentially causing a loss of function. Additional 4-bp and 5-bp deletions within *ycf15* led to internal stop codons in *Davidia involucrata* and *Hydrangea aspera*, respectively. Furthermore, in *Deutzia crassifolia,* a single substitution (G to A) within *ycf15* likewise resulted in an internal stop codon (TAG). In the remaining nine Cornales species, *ycf15* did not contain stop codons, and there was no evidence of loss of function (Fig. 4b). Because of these parallel function losses, *ycf15* was not annotated in this study.

### Phylogenetic analyses

The unfiltered whole plastid genome data set, with one copy of the IR region excluded, was 148,838 bp in length. Variable and parsimony informative sites of this data set were 25.5% and 15.5%, respectively. The noncoding regions were more variable than the coding regions (33.9% vs 17.5% variable sites and 20.3% vs 11.1% parsimony informative sites) (Table 3). Compared to the unfiltered alignment, a total of 22,247 sites (14.9%) in the lightly filtered data set and a total of 42,513 sites (28.6%) in the strictly filtered alignment were removed. The unfiltered and lightly filtered data sets showed similar percentages of variable and parsimony informative sites, irrespective of calculation for the different regions or the complete genome. However, the strictly filtered alignment exhibited a somewhat decreased percentage of variable and parsimony informative sites in the all data sets (Table 3).

**Table 3:** Sequence alignment information and support values for key nodes under different alignment strategies

Data set	Blocks	Number of sites	Variable sites	Parsimony informative sites	Support value (LB/PP)	
					(Cornaceae-Alangiaceae)-Curtisiaceae	(Mastixiaceae-Davidiaceae-Nyssaceae) - Hydrangeaceae
Coding	Unfiltered	75,334	13,147(17.5%)	8336(11.1%)	81/1.0	99/1.0
	Light filtered	74,352	13,040(17.5%)	8305(11.2%)	82/1.0	100/1.0
	Strict filtered	72,369	12,267(17.0%)	7809(10.8%)	78/1.0	100/1.0
Noncoding	Unfiltered	72,056	24,406(33.9%)	14,643(20.3%)	99/1.0	100/1.0
	Light filtered	51,343	18,974(37.0%)	12,232(23.8%)	99/1.0	100/1.0
	Strict filtered	32,852	10,167(30.9%)	6545(19.9%)	100/1.0	100/1.0
Complete	Unfiltered	148,838	37,928(25.5%)	23,136(15.5%)	100/1.0	100/1.0
	Light filtered	126,591	32,266(25.5%)	20,678(16.3%)	99/1.0	100/1.0
	Strict filtered	106,325	22,745(21.4%)	14,551(13.7%)	100/1.0	100/1.0

**Fig. 5:**
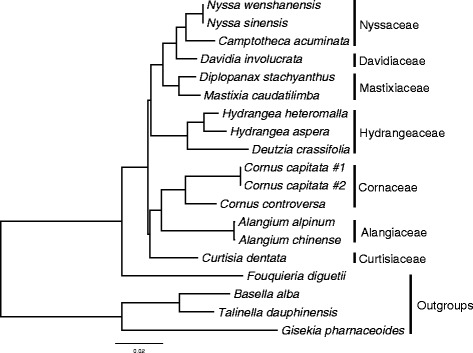
Phylogenetic relationships of Cornales based on unfiltered whole plastid genome alignment. Nodes without values represent maximal support in both ML and BI methods

Using both ML and BI methods without data partitioning, the phylogenetic inference of Cornales from the whole unfiltered data set provided complete resolution of relationships among all species sampled, with maximum support (100%/1.0) for all nodes (Fig. 5). Nyssaceae was monophyletic and sister in turn to Davidiaceae, then a monophyletic Mastixiaceae, then a monophyletic Hydrangeaceae. Cornales comprised this clade plus another, in which Cornaceae and Alangiaceae (both monophyletic) were together sister to Curtisiaceae (Fig. 5). The phylogenetic topology of Cornales based on unfiltered coding and noncoding regions were consistent with that from the complete plastome data set. Only the sister relationship of Curtisiaceae and Cornaceae-Alangiaceae received support values below 99% or 1.0 from unfiltered data sets, with 81%/1.0 support from coding regions (Table 3, Fig. 5, Additional file 2: Fig. S2).

Likewise, using lightly filtered and strictly filtered data sets, both the topology and support values were almost identical (Additional file 2: Fig. S3, S4). However, when only coding data sets are used, the bootstrap support value for the sister relationship of Curtisiaceae and Cornaceae-Alangiaceae drops to 78% with strictly filtered alignment (Additional file 2: Fig. S4A).

When partitioning was applied using the program PartitionFinder, the whole unfiltered data set was divided into 13 partitions (Table S3). Topology and support values obtained from this analysis were consistent with unpartitioned analysis, except for a decrease in BS support value from 100% to 95% for the sister relationship of Curtisiaceae to Cornaceae-Alangiaceae in ML analysis (Additional file 2: Fig. S2C).

## Discussion

### Structure of plastome and comparative analyses

In the present study, the complete plastid genomes of 14 species of Cornales were obtained for the first time. They showed the typical quadripartite structure of most angiosperms, including a pair of IR regions, separated by an LSC and an SSC region. The Cornales plastid genome was highly conserved in structure compared to most angiosperms [32], with all sampled species encoding the same set of 114 unique genes in same gene order (Table 2). The GC content was around the average for plant plastomes (GC = 37%) [60], but was slightly higher than that of the outgroup taxa. The length variation of the Cornales plastid genomes observed here was low (156–159 kpb), and differences were mainly due to variation in noncoding regions (65–68 kbp). Length variation of plastid genomes was previously shown to result from expansion and contraction of the inverted repeat regions [61]. Here, we also found that the IR/SSC boundary located differently among the 14 species, but the location of boundary and length of IR regions only showed moderate variation (Fig. 2). Furthermore, there was no obvious phylogenetic implication of extension/contraction of IRs among the Cornales plastomes.

### Premature stop codons in two genes, but no apparent loss of function in *rpl22*

The gene content is highly conserved among plastid genomes of land plants, although gene loss has been reported in several angiosperm lineages [17]. Two genes, *rpl22* and *ycf15,* contained premature termination codons in several species in the present study. *rpl22* showed premature termination codons in *Alangium*, *Cornus*, *Curtisia*, and *Mastixia*, making it about 20% shorter in these species compared to others. This gene appears to be absent from plastids in some taxa such as legumes [62] and was reported to have been transferred to the nucleus in both *Pisum* and Fagaceae [63, 64]. In Cornales, *rpl22* in those species with internal stop codons have not undergone a detectable increase in mutation rate compared to those without them, whereas such an increase would be expected if the gene was disabled in the former but functional in the latter. Moreover, the former group still contain nearly 80% of the normal gene sequence. This implies that *rpl22* in the plastid either functions as a gene in all examined species, or in none of them. If the former, truncation does not remove its function. If the latter, then it might be a pseudogene in all Cornales, as it is in *Citrus sinensis* [65]; if so it might have a non-coding function in the plastid. Possibly, a functional copy of *rpl22* might exist in the nucleus, as in *Pisum* and Fagaceae [63, 64], removing any selective disadvantage to loss of function in the chloroplast. Therefore, Cornales might be in the early stages of a process of losing *rpl22* from the plastid. Hence more data is needed, regarding function of *rpl22* in the chloroplast and whether a full copy exists in the nucleus.

The nucleotide sequence of *ycf15* has been shown to vary among angiosperm plastid genomes, with conserved motifs at 5′ to 3′ ends in some taxa (like tobacco) and an intervening region of about 250 bp in some other taxa (like *Eucalyptus globulus*) that renders it as a pseudogene [66]. A comparative study of *ycf15* transcripts in taxa with or without the insertion suggested that this gene may not be a protein-coding gene even when it is intact [67]. Although transcripts of *ycf15* were detected in some taxa like *Camellia*, it may have been removed from the pre-mRNA after transcription in order to activate the function of other genes, thus *ycf15* is possibly an intergenic sequence without function [68]. The non-coding *ycf15* hypothesis to some extent is supported by data from Cornales, within which four independent mutation events within *ycf15* either inserted stop codons (*Davidia, Hydrangea* and *Deutzia*) or deleted parts of the gene (*Alangium*). The evolutionary patterns of *ycf15* showed that they evolved in a discontinuous fashion across angiosperms [68] [69]. It shows an intact and conserved structure in nine Cornales species, but cannot be translated normally in species of *Alangium*, *Davidia* and *Deutzia*. These three genera belong to distinct clades, implying separate and independent alterations in each case; hence *ycf15* might not provide phylogenetic implication (Fig. 5).

### Influence of data set subdivision, alignment filtering, and data partitioning on phylogeny

In addition to the complete plastid genome, two data subsets were generated, one comprising all coding genes, and the other only noncoding regions. We conducted three filtering strategies (none, light and strict) on each of these three data sets. Phylogenetic inference from BI and ML analyses based on all data sets provided the same topology. All data sets supported the sister relationship between Curtisiaceae and Cornaceae-Alangiaceae, support for this clade from the coding region and its filtered data subsets was relatively low, e.g. 81%/1.0 for unfiltered (Additional file 2: Fig. S2A). Although the noncoding regions are usually excluded for phylogenomic analyses at high taxonomic levels [14, 24], the phylogenetic resolution within Cornales obtained from noncoding regions in all three strategies was high (Additional file 2: Fig. S2, S3, S4; Table 3). This might because plastid genomes within the order have a conserved collinear structure, and the noncoding regions can provide more phylogenetic signals. The treatment of problematic or ambiguous regions in alignments can affect the final phylogenetic relationships, and for alignments that are long enough, removal of problematic regions leads to better phylogenetic resolution [69, 70]. Conversely, in this study, alignment filtering has no influence in any of the coding, noncoding and complete alignment data sets (Table 3), which may also be due to the conservation of plastid genomes within Cornales.

When a genome-scale approach is adopted in phylogenetic analyses, partitioning is one of the most popular methods used to model the heterogeneity of molecular evolution among regions in an alignment for phylogenetic inference [71]. In the present study, however, data partitioning by PartitionFinder had no effect on the topology of the resulting phylogenetic trees compared to unpartitioned plastid genome data set. The phylogenetic relationships of Cornales were robustly resolved based on both partitioned and unpartitioned datasets. It was indicated that the longer the data set was, the less likely that the results will be affected by partitioning scheme [71]. This is perhaps because the whole plastid genome contains sufficient amount of phylogenetic signals (while noise is randomly dispersed) and may converge on the correct phylogenetic tree, irrespective of partitioning. It was also observed, in a previous phylogenetic study of Cornales with six plastid fragments, that the partitioned data sets presented the same topology as the unpartitioned ones with only some differences among the branch support values [3]. Irrespective of different regions or data subsets, partitioned or unpartitioned data sets used, our results suggested that the plastid genome as a whole contains sufficient phylogenetic signals in different regions within Cornales to fully resolve the phylogenetic relationships. The conservatism in genome structure and gene content along with abundance of phylogenetic signal of Cornales plastid genomes, both coding and noncoding regions, make it a valuable phylogenetic tool, at and below family level.

### Phylogenetic implication among Cornales with plastid genome

Preliminary phylogenetic frameworks for Cornales have previously been provided based on a few molecular markers, but relationships among families of the order tended to be poorly resolved [5, 7, 72]. A later study [3], based on six plastid loci (*rbcL*, *matK*, *ndhF*, *atpB*, *trnL-F* and *trnH-K*) and a broader taxon sampling, recovered five major clades: Cornaceae-Alangiaceae, Curtisiaceae-Grubbiaceae, Mastixiaceae-Nyssaceae-Davidiaceae, Hydrostachyaceae, and Hydrangeaceae-Loasaceae; relationships between these clades were well supported. However, cpDNA-based relationships were contradicted by 26S rDNA data in the study, and relationships within some families (e.g. Hydrangeaceae, Cornaceae) were not fully recovered [3]. Therefore, further work is needed on relationships within these families. Our own work recovered identical relationships with even higher support, demonstrating that greater genome coverage can compensate for reduced taxon sampling, at least in some cases. The sequence and structure of the whole plastid genome has been recognized for its great potential to resolve relationships for phylogenetically recalcitrant plant groups [14, 21, 40, 73, 74]. Given that resources will seldom permit full genome sequencing across large numbers of taxa, the best strategy for wide taxonomic sampling is to identify marker regions that contain a high proportion of phylogenetically useful information. To this end, our study identified two genes (*matK*, *ndhF*) and seven regions *trnK-rps16*, *rpoB-trnC*, *trnT-trnE*, *petA-psbJ*, *psbE-petL*, *rpl32-trnL*, and *rps15-ycf1* that are mutational hotspots, and are hence recommended as phylogenetic markers within Cornales, and perhaps beyond it.

## Conclusions

Phylogenomic data have rapidly accumulated and been broadly used for resolving phylogenetic relationships in the last few years. In the present study, fifteen full plastid genomes of 14 Cornales species were sequenced to investigate the phylogenetic relationships and plastome evolution of Cornales. Comparative analysis of the plastid genomes revealed that plastomes of the order have a conserved collinear structure with identical gene content and order. Two genes (*rpl22* and *ycf15*) contained premature stop codons in seven and five species respectively. Plastid genomes showed strong potential for resolving phylogenetic relationships within Cornales, both for the interfamily and intrafamily relationships, with very strong support. Different partitioning schemes and filtering strategies (none, light and strict) of sequence data sets have no effect on phylogenetic relationships. The topology recovered from coding and noncoding data sets was likewise identical to that for the whole plastome. However, the coding data set provided lower support values than the latter two data sets. Mutational hotspots and highly informative regions of Cornales were identified. All data presented here are fundamental to phylogenomic analyses of Cornales, and will be a useful genomic resource for future studies of evolutionary biology.

## Additional files

Additional file 1: Table S1. The primers newly designed in this study for four junctions and two genes (*rpl22*, *ycf15*). **Table S2**. Molecular models selected for all the data sets of the three alignment strategies. **Table S3**. Model selected for each data partition identified by software PartitionFinder for unfiltered complete plastid genomes. **Table S4**. Gene category and gene contained in plastid genomes of Cornales. (DOC 147 kb)

Additional file 2: Fig. S1. A percent identity plot showing the overall sequence similarity of the fourteen Cornales plastid genomes. **Fig. S2**. Phylogenetic relationships of Cornales based on three different data sets with light filtered alignment. **Fig. S3**. Phylogenetic relationships of Cornales based on three different data sets with strict filtered alignment. (PDF 1121 kb)

## Abbreviations

AIC: Akaike information criterion
BI: Bayesian inference
BIC: Bayesian information criterion
GC: Guaninecytosine
IR: Inverted repeat region
LB: Likelihood bootstrap
LSC: Large single copy region
ML: Maximum likelihood
PP: Posterior probability
SSC: Small single region
