# Supplementary material for: Comparative analyses of plastid genomes from fourteen Cornales species: inferences for phylogenetic relationships and genome evolution
Source: BMC Genomics. 2017 Dec 8;18:956. doi: 10.1186/s12864-017-4319-9 (PMC5721659; doi:10.1186/s12864-017-4319-9)
Supplement: Supplementary file 1 — The primers newly designed in this study for four junctions and two genes (rpl22, ycf15). Table S2. Molecular models selected for all the data sets of the three alignment strategies. Table S3. Model selected for each data partition identified by software PartitionFinder for unfiltered complete plastid genomes. Table S4. Gene category and gene contained in plastid genomes of Cornales. (DOC 147 kb) [file 12864_2017_4319_MOESM1_ESM.doc]

**Table S1.** The primers newly designed in this study for four junction and two genes (*rpl22*, *ycf15*).

| Taxon | Sequence (5’>3’) |
| --- | --- |
| *Alangium alpinum* | F TCGGAAGAAAAGGAGGATCCG  R GGGCGCATCTCTTCTTATCT |
| *Alangium alpinum* | F TGATTCTTCGTCGCCGTAGT  R CCGTGCTAACCTTGGTATGGA |
| *Alangium alpinum* | F AGGGAATGATGACCTTGGGTT  R TGATTCTTCGTCGCCGTAGT |
| *Alangium alpinum* | F TCAAGAAAGGATCATACATTTTAGGCA  R CGGAAGAAAAGGAGGATCCGG |
| *Alangium alpinum* | F AGCCGGAAAGATATGTGGCC  R GGGATTGGCGACTTACCCAT |
| *Alangium chinense* | F TCGGAAGAAAAGGAGGATCCG  R GGGCGCATCTCTTCTTATCT |
| *Alangium chinense* | F TGATTCTTCGTCGCCGTAGT  R CCGTGCTAACCTTGGTATGGA |
| *Alangium chinense* | F AGGGAATGATGACCTTGGGTT  R TGATTCTTCGTCGCCGTAGT |
| *Alangium chinense* | F TCAAGAAAGGATCATACATTTTAGGCA  R CGGAAGAAAAGGAGGATCCGG |
| *Alangium chinense* | F AGCCGGAAAGATATGTGGCC  R GGGATTGGCGACTTACCCAT |
| *Camptotheca acuminata* | F TCGGAAGAAAAGGAGGATCCG  R GGGCGCATCTCTTCTTATCT |
| *Camptotheca acuminata* | F TGATTCTTCGTCGCCGTAGT  R CCGTGCTAACCTTGGTATGGA |
| *Camptotheca acuminata* | F AGGGAATGATGACCTTGGGTT  R TGATTCTTCGTCGCCGTAGT |
| *Camptotheca acuminata* | F TCAAGAAAGGATCATACATTTTAGGCA  R CGGAAGAAAAGGAGGATCCGG |
| *Camptotheca acuminata* | F AGCCGGAAAGATATGTGGCC  R GGGATTGGCGACTTACCCAT |
| *Cornus capitata* | F TCGGAAGAAAAGGAGGATCCG  R GGGCGCATCTCTTCTTATCT |
| *Cornus capitata* | F TGATTCTTCGTCGCCGTAGT  R CCGTGCTAACCTTGGTATGGA |
| *Cornus capitata* | F AGGGAATGATGACCTTGGGTT  R TGATTCTTCGTCGCCGTAGT |
| *Cornus capitata* | F TCAAGAAAGGATCATACATTTTAGGCA  R CGGAAGAAAAGGAGGATCCGG |
| *Cornus capitata* | F AGCCGGAAAGATATGTGGCC  R GGGATTGGCGACTTACCCAT |
| *Cornus controversa* | F TCGGAAGAAAAGGAGGATCCG  R GGGCGCATCTCTTCTTATCT |
| *Cornus controversa* | F TGATTCTTCGTCGCCGTAGT  R CCGTGCTAACCTTGGTATGGA |
| *Cornus controversa* | F AGGGAATGATGACCTTGGGTT  R TGATTCTTCGTCGCCGTAGT |
| *Cornus controversa* | F TCAAGAAAGGATCATACATTTTAGGCA  R CGGAAGAAAAGGAGGATCCGG |
| *Cornus controversa* | F AGCCGGAAAGATATGTGGCC  R GGGATTGGCGACTTACCCAT |
| *Curtisia dentata* | F TCGGAAGAAAAGGAGGATCCG  R GGGCGCATCTCTTCTTATCT |
| *Curtisia dentata* | F TGATTCTTCGTCGCCGTAGT  R CCGTGCTAACCTTGGTATGGA |
| *Curtisia dentata* | F AGGGAATGATGACCTTGGGTT  R TGATTCTTCGTCGCCGTAGT |
| *Curtisia dentata* | F TCAAGAAAGGATCATACATTTTAGGCA  R CGGAAGAAAAGGAGGATCCGG |
| *Curtisia dentata* | F AGCCGGAAAGATATGTGGCC  R GGGATTGGCGACTTACCCAT |
| *Deutzia crassifolia* | F TCGGAAGAAAAGGAGGATCCG  R GGGCGCATCTCTTCTTATCT |
| *Deutzia crassifolia* | F TGATTCTTCGTCGCCGTAGT  R CCGTGCTAACCTTGGTATGGA |
| *Deutzia crassifolia* | F AGGGAATGATGACCTTGGGTT  R TGATTCTTCGTCGCCGTAGT |
| *Deutzia crassifolia* | F TCAAGAAAGGATCATACATTTTAGGCA  R CGGAAGAAAAGGAGGATCCGG |
| *Deutzia crassifolia* | F AGCCGGAAAGATATGTGGCC  R GGGATTGGCGACTTACCCAT |
| *Diplopanax stachyanthus* | F TCGGAAGAAAAGGAGGATCCG  R GGGCGCATCTCTTCTTATCT |
| *Diplopanax stachyanthus* | F TGATTCTTCGTCGCCGTAGT  R CCGTGCTAACCTTGGTATGGA |
| *Diplopanax stachyanthus* | F AGGGAATGATGACCTTGGGTT  R TGATTCTTCGTCGCCGTAGT |
| *Diplopanax stachyanthus* | F TCAAGAAAGGATCATACATTTTAGGCA  R CGGAAGAAAAGGAGGATCCGG |
| *Diplopanax stachyanthus* | F AGCCGGAAAGATATGTGGCC  R GGGATTGGCGACTTACCCAT |
| *Davidia involucrata* | F TCGGAAGAAAAGGAGGATCCG  R GGGCGCATCTCTTCTTATCT |
| *Davidia involucrata* | F TGATTCTTCGTCGCCGTAGT  R CCGTGCTAACCTTGGTATGGA |
| *Davidia involucrata* | F AGGGAATGATGACCTTGGGTT  R TGATTCTTCGTCGCCGTAGT |
| *Davidia involucrata* | F TCAAGAAAGGATCATACATTTTAGGCA  R CGGAAGAAAAGGAGGATCCGG |
| *Davidia involucrata* | F AGCCGGAAAGATATGTGGCC  R GGGATTGGCGACTTACCCAT |
| *Hydrangea heteromalla* | F TCGGAAGAAAAGGAGGATCCG  R GGGCGCATCTCTTCTTATCT |
| *Hydrangea heteromalla* | F TGATTCTTCGTCGCCGTAGT  R CCGTGCTAACCTTGGTATGGA |
| *Hydrangea heteromalla* | F AGGGAATGATGACCTTGGGTT  R TGATTCTTCGTCGCCGTAGT |
| *Hydrangea heteromalla* | F TCAAGAAAGGATCATACATTTTAGGCA  R CGGAAGAAAAGGAGGATCCGG |
| *Hydrangea heteromalla* | F AGCCGGAAAGATATGTGGCC  R GGGATTGGCGACTTACCCAT |
| *Hydrangea aspera* | F TCGGAAGAAAAGGAGGATCCG  R GGGCGCATCTCTTCTTATCT |
| *Hydrangea aspera* | F TGATTCTTCGTCGCCGTAGT  R CCGTGCTAACCTTGGTATGGA |
| *Hydrangea aspera* | F AGGGAATGATGACCTTGGGTT  R TGATTCTTCGTCGCCGTAGT |
| *Hydrangea aspera* | F TCAAGAAAGGATCATACATTTTAGGCA  R CGGAAGAAAAGGAGGATCCGG |
| *Hydrangea aspera* | F AGCCGGAAAGATATGTGGCC  R GGGATTGGCGACTTACCCAT |
| *Mastixia caudatilimba* | F TCGGAAGAAAAGGAGGATCCG  R GGGCGCATCTCTTCTTATCT |
| *Mastixia caudatilimba* | F TGATTCTTCGTCGCCGTAGT  R CCGTGCTAACCTTGGTATGGA |
| *Mastixia caudatilimba* | F AGGGAATGATGACCTTGGGTT  R TGATTCTTCGTCGCCGTAGT |
| *Mastixia caudatilimba* | F TCAAGAAAGGATCATACATTTTAGGCA  R CGGAAGAAAAGGAGGATCCGG |
| *Mastixia caudatilimba* | F AGCCGGAAAGATATGTGGCC  R GGGATTGGCGACTTACCCAT |
| *Nyssa wenshanensis* | F TCGGAAGAAAAGGAGGATCCG  R GGGCGCATCTCTTCTTATCT |
| *Nyssa wenshanensis* | F TGATTCTTCGTCGCCGTAGT  R CCGTGCTAACCTTGGTATGGA |
| *Nyssa wenshanensis* | F AGGGAATGATGACCTTGGGTT  R TGATTCTTCGTCGCCGTAGT |
| *Nyssa wenshanensis* | F TCAAGAAAGGATCATACATTTTAGGCA  R CGGAAGAAAAGGAGGATCCGG |
| *Nyssa wenshanensis* | F AGCCGGAAAGATATGTGGCC  R GGGATTGGCGACTTACCCAT |
| *Fouquieria diguetii* | F TCGGAAGAAAAGGAGGATCCG  R GGGCGCATCTCTTCTTATCT |
| *Fouquieria diguetii* | F TGATTCTTCGTCGCCGTAGT  R CCGTGCTAACCTTGGTATGGA |
| *Fouquieria diguetii* | F AGGGAATGATGACCTTGGGTT  R TGATTCTTCGTCGCCGTAGT |
| *Fouquieria diguetii* | F TCAAGAAAGGATCATACATTTTAGGCA  R CGGAAGAAAAGGAGGATCCGG |
| *Fouquieria diguetii* | F AGCCGGAAAGATATGTGGCC  R GGGATTGGCGACTTACCCAT |
| *Basella alba* | F TGCAGTTTTAGACAAAGGACTTTT  R TGTCTAATTTCGGTTGATAGGGA |
| *Basella alba* | F AGAGCCGGATCTAAGCGTTG  R GCGGATGTAGCCAAGTGGAT |
| *Basella alba* | F GCAATTCCTTCGACACCTGA  R GGGGTTATCCTGCACTTGGA |
| *Basella alba* | F GATGTTGATTCCCCGGCGAA  R GGAATCGTCCAACGCGATAT |
| *Basella alba* | F AGCCGGAAAGATATGTGGCC  R GGGATTGGCGACTTACCCAT |
| *Gisekia pharnaceoides* | F ACCCAGTGTATCAATTTTCTGGGA  R ATAGGAGGGGGTCGCATTTC |
| *Gisekia pharnaceoides* | F AGAGCCGGATCTAAGCGTTG  R GCGGATGTAGCCAAGTGGAT |
| *Gisekia pharnaceoides* | F GCAATTCCTTCGACACCTGA  R GGGGTTATCCTGCACTTGGA |
| *Gisekia pharnaceoides* | F GATGTTGATTCCCCGGCGAA  R GGAATCGTCCAACGCGATAT |
| *Gisekia pharnaceoides* | F AGCCGGAAAGATATGTGGCC  R GGGATTGGCGACTTACCCAT |
| *Talinella dauphinensis* | F AGCCATTTTCTGGGAAATGATACA  R GGAGGAGGTCGCATTTCCTC |
| *Talinella dauphinensis* | F AGGAGTAATTAACTGTGACACGT  R AAATTGGAAAGGGGGCGGAT |
| *Talinella dauphinensis* | F ACCAAGTCTGAAACCAAGTGGA  R AGGAGTAATTAACTGTGACACGT |
| *Talinella dauphinensis* | F ATGTTGATTCCCCGGCGAAT  R AGCCATTTTCTGGGAAATGATACA |
| *Talinella dauphinensis* | F TGTGGCCATGAAAGGAGGAT  R TGGCACTGTACGTTCCCAAA |

**TableS2.** Molecular models selected for all the dataset of the three alignment strategies.

| Data set | Blocks | Best fit model | ML | BI |
| --- | --- | --- | --- | --- |
|  |  |  |  |  |
| Coding | Unfiltered | TVM+I+G | GTR+G | GTR+I+G |
|  | Light filtered | TVM+I+G | GTR+G | GTR+I+G |
|  | Strict filtered | TVM+I+G | GTR+G | GTR+I+G |
| Noncoding | Unfiltered | GTR+G | GTR+G | GTR+G |
|  | Light filtered | GTR+I+G | GTR+G | GTR+I+G |
|  | Strict filtered | TVM+I+G | GTR+G | GTR+I+G |
| Complete | Unfiltered | TVM+I+G | GTR+G | GTR+I+G |
|  | Light filtered | GTR+I+G | GTR+G | GTR+I+G |
|  | Strict filtered | GTR+I+G | GTR+G | GTR+I+G |

**TableS3.** Model selected for each data partition identified by software PartitionFinder for unfiltered complete plastid genomes.

| Partition | Partition identity | Model selection |
| --- | --- | --- |
| 1 | rRNA, tRNA | GTR+I+G |
| 2 | *trnH*(GUG)-*psbA* | GTR+G |
| 3 | *accD, atpA, atpB, atpE, atpF, atpI, cemA, clpP, infA, ndhA, ndhC, ndhD, ndhE, ndhG, ndhH, ndhI, ndhJ, ndhK, petA, petB, petD, psaA, psaB, psaC, psaI_psaJ, psbA, psbB-psbT-psbN, psbC, psbD, psbH, psbK-psbI, rbcL, rpl14, rpl16, rpl20, rpl20-rps12, rps12-clpP, rpl2-intron, rpl33, rpoA, rpoA-rps11,rps11-rpl36,rpl36-infA,infA-rps8, rpoB, rpoC1, rpoC2, rpoC2-rpoC1, rps11, rps12, rps14, rps16, rps2, rps4, rps8, trnR*(*ACG*)*-trnN*(*GUU*)*, trnS*(*UGA*)*-lhbA, trnV*(*UAC*)*-trnM*(*CAU*)*, trnV*(*UAC*)*-intron, trnfM*(*CAU*)*-rps14, rps14-psaB, psaB-psaA, ycf3-intron1, ycf4* | GTR+I+G |
| 4 | *atpB-rbcL, atpI-rps2, cemA-petA, clpP-intron1, clpP-intron2, lhbA-trnG*(*UCC*)*, matK-trnK*(*UUU*)*, ndhA-intron, ndhJ-ndhK, ndhK-ndhC, petB-petD, petB-intron, petD-rpoA, petD-intron, petL-petG_petG-trnW*(*CCA*)*, petN-psbM, psaI-ycf4, psbA-trnK*(*UUU*)*, psbB-psbT, psbC-trnS*(*UGA*)*, rbcL-accD, rpl16-intron, rpl36_rpl32, rps16-intron, rps2-rpoC2, trnK*(*UUU*)*-matK, trnL*(*UAA*)*-intron, trnQ*(*UUG*)*-psbK* | GTR+G |
| 5 | *atpF-intron, ccsA, clpP-psbB, matK, psbK-psbI, psbM_ihbA, psbT-psbN, psbN-psbH, psbH-petB, rpoC1-intron, rps15, rps18, rps19, rps3, rps8-rpl14, rpl14-rpl16, trnG*(*GCC*)*-intron, ycf3-intron2* | GTR+G |
| 6 | *ndhF*-*rpl32*, *trnE*(UUU)-*trnT*(GGU), *trnK*(UUU)-*rps16* | GTR+G |
| 7 | *ndhC-trnV*(*UAC*)*, ndhG-ndhI, rpl22, rpl32-trnL*(*UAG*)*, rpl33-rps18, rps15-ycf1, rps16-trnQ*(*UUG*)*, rps4-trnT*(*UGU*)*, trnG*(*GCC*)*-trnR*(*UCU*)*, trnG*(*UCC*)*-trnfM*(*CAU*)*, trnL*(*UAG*)*-ccsA, ccsA-ndhD, ndhD-psaC, trnS*(*GCU*)*-trnG*(*GCC*)*, trnT*(*UGU*)*-trnL*(*UAA*) | GTR+G |
| 8 | *accD-psaI, atpF-atpH, atpH-atpI, ndhI-ndhA, ndhA-ndhH, ndhH-rps15, petA-psbJ, psbJ-psbL, psbL-psbF, psbF-psbE, psaA-ycf3, psaC-ndhE, psaJ-rpl33, psbE-petL, psbM-trnD*(*GUC*)*, rpl16-rps3_rpl22-rps19_rps19-rpl2, rpoC1-rpoB-rpoB-trnC*(*GCA*)*, rps18-rpl20, trnC*(*GCA*)*-petN, trnD*(*GUC*)*-trnY, trnY-trnE*(*UUU*)*, trnF*(*GAA*)*-ndhJ, trnL*(*UAA*)*-trnF*(*GAA*)*, trnM*(*CAU*)*-atpE, trnP*(*UGG*)*-psaJ, trnR*(*UCU*)*-atpA, atpA-atpF, trnS*(*GGA*)*-rps4, trnT*(*GGU*)*-psbD, trnW*(*CCA*)*-trnP*(*UGG*)*, ycf3-trnS*(*GGA*)*, ycf4-cemA* | GTR+G |
| 9 | *psbI*-*trnS*(GCU) | GTR+G |
| 10 | *16SrRNA-trnI*(*GAU*)*, 4.5SrRNA-5SrRNA, 5SrRNA-trnR*(*ACG*)*, atpH, ndhB, ndhB-rps7, rps7-rps12, ndhB-intron, petL_petG, petG-petN, psbJ_psbL, psbL-psbF, psbF-psbE, rpl2, rpl23, rpl2-rpl23, rpl23-trnI*(*CAU*)*, trnI*(*CAU*)*-ycf2, ycf2-ycf15, rps12-intron1, rps12-trnV*(*GAC*)*, rps7, trnA*(*UGC*)*-intron, trnI*(*GAU*)*-trnA*(*UGC*)*, trnA*(*UGC*)*-23SrRNA, 23SrRNA-4.5SrRNA, trnI*(*GAU*)*-intron, trnL*(*CAA*)*-ndhB, trnN*(*GUU*)*-ycf1like, trnV*(*GAC*)*-16SrRNA, ycf15, ycf15-trnL*(*CAA*)*, ycf2, ycf3* | GTR+G |
| 11 | *trnN*(*GUU*)*-ndhF* | GTR+I+G |
| 12 | *ndhF* | GTR+I+G |
| 13 | *ndhE-ndhG, ycf1* | GTR+ G |

**TableS4. Gene category and gene contained in plastid genomes of Cornales.**

| Category | Group of gene | Name of gene |
| --- | --- | --- |
| Self replication | Ribosomal RNA genes | *rrn4.5*(×2), *rrn5*(×2), *rrn16*(×2), *rrn23(×2)* |
|  | Transfer RNA genes | *trnA-UGC**(×2), *trnC-GCA*, *trnD-GUC*, *trnE-UUC*, *trnF-GAA*, *trnfM-CAU*, *trnG-GCC**, *trnG-UCC*, *trnH-GUG*, *trnI-CAU*(×2), *trnI-GAU**(×2), *trnK-UUU**, *trnL-CAA*(×2), *trnL-UAA**, *trnL-UAG*, *trnM-CAU*, *trnN-GUU*(×2), *trnP-GGG*, *trnP-UGG*, *trnQ-UUG*, *trnR-ACG*(×2), *trnR-UCU*, *trnS-GCU*, *trnS-GGA*, *trnS-UGA*, *trnT-GGU*, *trnT-UGU*, *trnV-GAC*(×2), *trnV-UAC**, *trnW-CCA*, *trnY-GUA* |
|  | Small subunit of ribosome | *rps2*, *rps3*, *rps4*, *rps7*(×2), *rps8*, *rps11*, *rps12*(×2*, *part)*, *rps14*, *rps15*, *rps16**, *rps18* , *rps19* |
|  | Large subunit of ribosome | *rpl2**(×2), *rpl14*, *rpl16**, *rpl20*, *rpl22*(in 8 individuals), *rpl23*(×2), *rpl32*, *rpl33*, *rpl36* |
|  | RNA polymerasesubunits | *rpoA*, *rpoB*, *rpoC1**, *rpoC2* |
| Photosynthesis | NADH dehydrogenase | *ndhA**, *ndhB**(×2), *ndhC*, *ndhD*, *ndhE*, *ndhF*, *ndhG*, *ndhH*, *ndhI*, *ndhJ*, *ndhK* |
|  | Photosystem Ⅰ | *psaA*, *psaB*, *psaC*, *psaI*, *psaJ*, *ycf3*** |
|  | Photosystem Ⅱ | *psbA*, *psbB*, *psbC*, *psbD*, *psbE*, *psbF*, *psbH*,*psbI*, *psbJ*, *psbK*, *psbL*, *psbM*, *psbN*, *psbT*, *psbZ* |
|  | Cytochrome b/f complex | *petA*, *petB**, *petD**, *petG*, *petL*, *petN* |
|  | ATP synthase | *atpA*, *atpB*, *atpE*, *atpF**, *atpH*, *atpI* |
|  | Large subunit of rubisco | *rbcL* |
| Other genes | Translational initiation factor | *infA* |
|  | Maturase | *matK* |
|  | Protease | *clpP*** |
|  | Envelope membrane protein | *cemA* |
|  | Subunit of acetyl-CoA-carboxylase | *accD* |
|  | c-type cytochrome synthesis gene | *ccsA* |
| Unknown gene | Conserved open reading frames | *ycf1*(×2,part), *ycf2*(×2), *ycf4* |

Note: one and two asterisks indicate one-intron and two-intron containing genes, respectively. Genes located in the IR region are indicated by (×2).
