# Supplementary material for: Comparative analyses of plastid genomes from fourteen Cornales species: inferences for phylogenetic relationships and genome evolution
Source: BMC Genomics. 2017 Dec 8;18:956. doi: 10.1186/s12864-017-4319-9 (PMC5721659; doi:10.1186/s12864-017-4319-9)
Supplement: Supplementary file 2 — A percent identity plot showing the overall sequence similarity of the fourteen Cornales plastid genomes. Fig. S2. Phylogenetic relationships of Cornales based on three different data sets with light filtered alignment. Fig. S3. Phylogenetic relationships of Cornales based on three different data sets with strict filtered alignment. (PDF 1121 kb) [file 12864_2017_4319_MOESM2_ESM.pdf]

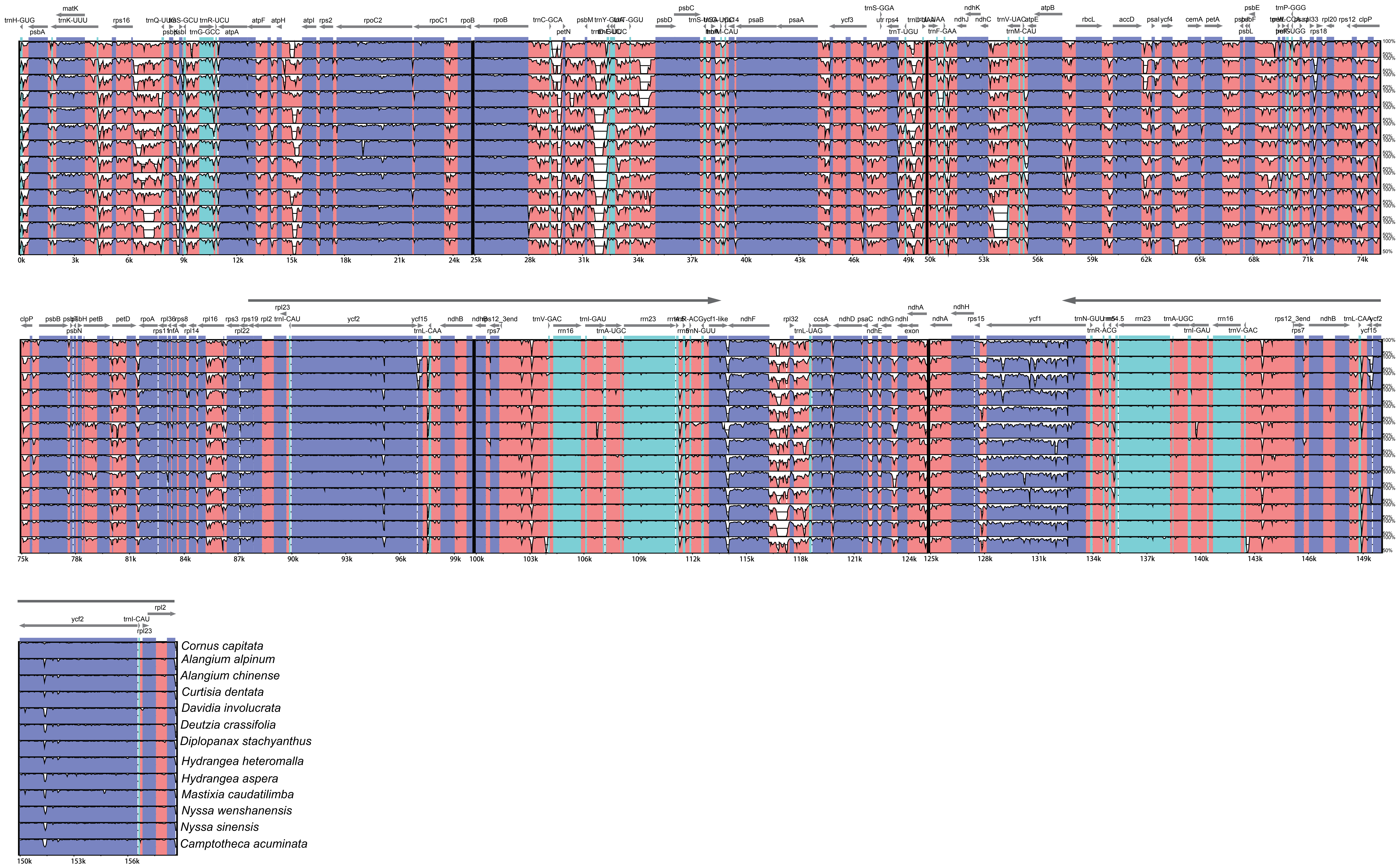

Figure S1. A percent identity plot showing the overall sequence similarity of the fourteen Cornales plastid genomes. Pair-wise comparison of plastid genomes within the Cornales with *Cornus controversa* as a reference in the mVISTA program. The Y scale represents % identity ranging from 50–100%. Genome regions are color-coded as protein coding, tRNA or rRNA genes, and conserved noncoding sequences.

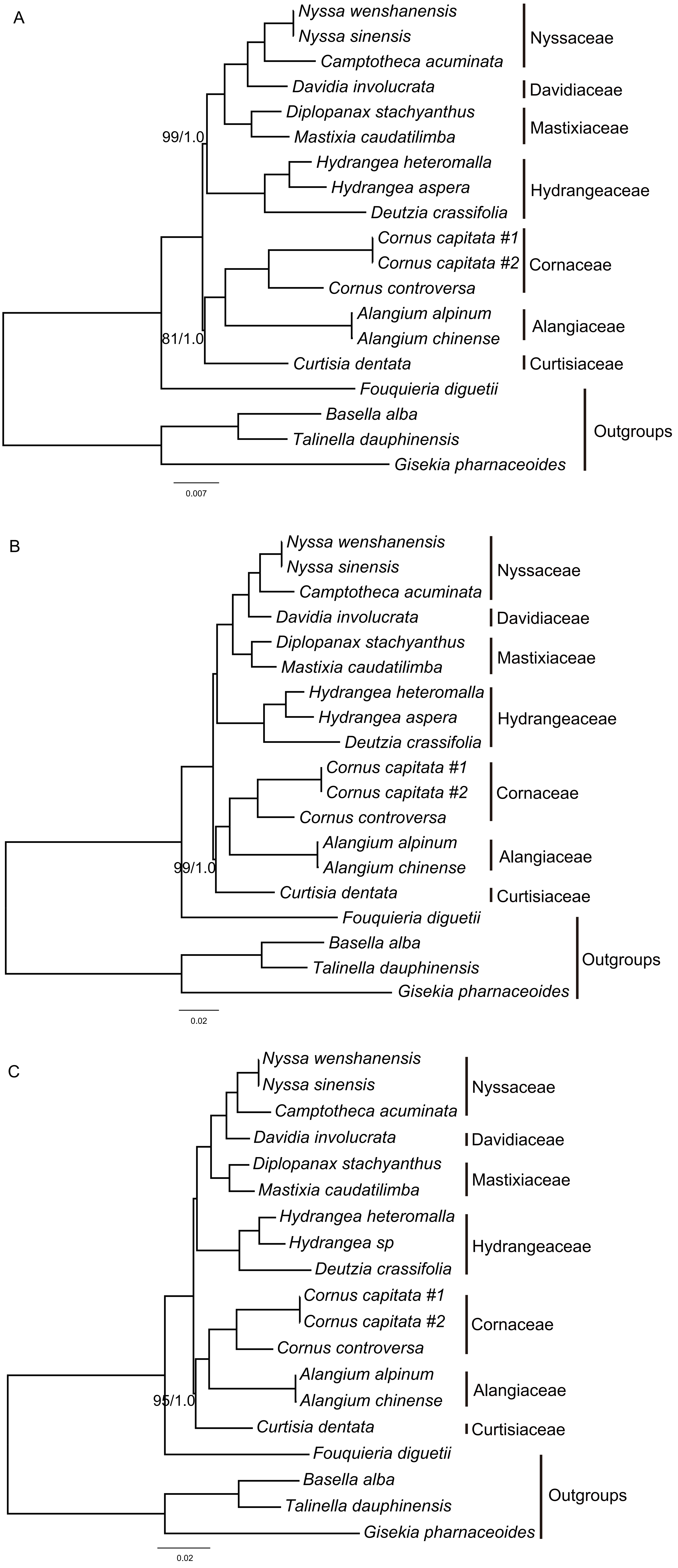

Figure S2. Phylogenetic relationships of Cornales based on three different data sets with unfiltered alignment. (A) Coding genes; (B) Noncoding regions; (C) Partitioned plastid genome. In each case the ML tree is shown, with maximum likelihood bootstrap (LB)/Bayesian inference posterior probability (PP) values given for each node. Nodes without values represent maximal support in both analyses.

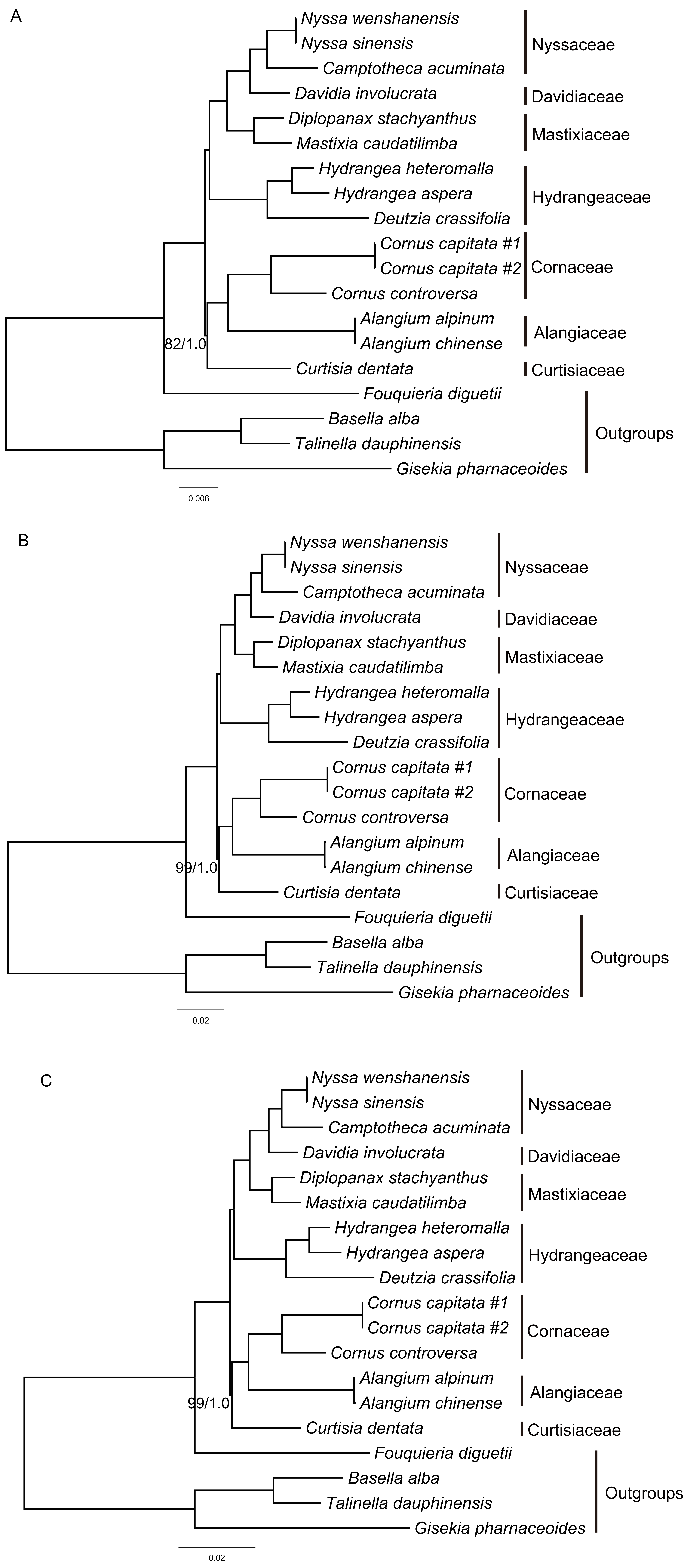

Figure S3. Phylogenetic relationships of Cornales based on three different data sets with light filtered alignment. (A) Coding genes; (B) Noncoding regions; (C) Whole plastid genome. In each case the ML tree is shown, with maximum likelihood bootstrap (LB)/Bayesian inference posterior probability (PP) given for nodes. Nodes with no support values represent maximal support values in both analyses.

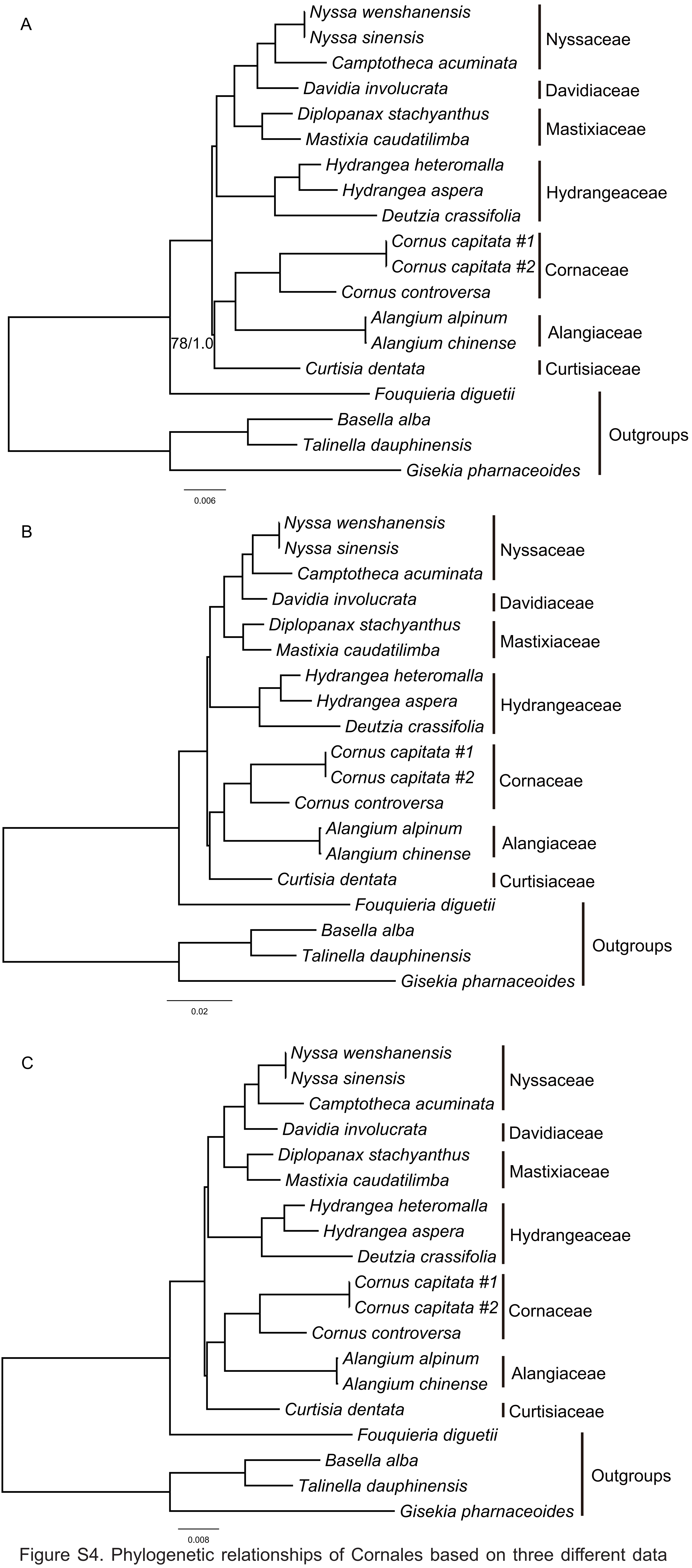

Figure S4. Phylogenetic relationships of Cornales based on three different data sets with strict filtered alignment. (A) Common coding genes; (B) Noncoding regions; (F) Whole plastid genome. In each case the ML tree is shown, with maximum likelihood bootstrap (LB)/Bayesian inference posterior probability (PP) given for nodes. Nodes with no support values represent maximal support values in both analyses.
